# Inhibition of the aquaporin 3 water channel increases the sensitivity of prostate cancer cells to cryotherapy

**DOI:** 10.1038/sj.bjc.6605093

**Published:** 2009-06-09

**Authors:** M Ismail, S Bokaee, J Davies, K J Harrington, H Pandha

**Affiliations:** 1 Department of Oncology, Postgraduate Medical School, University of Surrey, Guildford GU2 7WG, UK; 2 Department of Urology, The Royal Surrey County Hospital, Guildford GU2 7XX UK; 3 Targeted Therapy Laboratory, The Institute of Cancer Research, 237 Fulham Road, London SW3 6JB, UK

**Keywords:** AQP3, cryotherapy, prostate cancer

## Abstract

Aquaporins (*AQPs*) are intrinsic membrane proteins that facilitate selective water and small solute movement across the plasma membrane. In this study, we investigate the role of inhibiting *AQPs* in sensitising prostate cancer cells to cryotherapy. PC-3 and DU145 prostate cancer cells were cooled to 0, −5 and −10°C. The expression of *AQP3* in response to freezing was determined using real-time quantitative polymerase chain reaction (RT–qPCR) and western blot analysis. Aquaporins were inhibited using mercuric chloride (HgCl_2_) and small interfering RNA (siRNA) duplex, and cell survival was assessed using a colorimetric assay. There was a significant increase in *AQP3* expression in response to freezing. Cells treated with *AQP3* siRNA were more sensitive to cryoinjury compared with control cells (*P*<0.001). Inhibition of the AQPs by HgCl_2_ also increased the sensitivity of both cell lines to cryoinjury and there was a complete loss of cell viability at −10°C (*P*<0.01). In conclusion, we have shown that *AQP3* is involved directly in cryoinjury. Inhibition of *AQP3* increases the sensitivity of prostate cancer cells to freezing. This strategy may be exploited in the clinic to improve the efficacy of prostate cryotherapy.

Prostate cancer is recognised as one of the most common non-dermatological male cancers in the United Kingdom, accounting for almost one in four of all new male cancers (Cancer research UK, 2007). It represents the second most common cause of cancer death (Jemal *et al*, 2007). Radical prostatectomy and external beam radiotherapy remain the two main active treatment modalities for prostate cancer with acceptable results, although they may be associated with varying degrees of morbidity (Lim *et al*, 1995; Kupelian *et al*, 1997; Aus *et al*, 2005). Prostate cryotherapy is the localised application of a freezing temperature resulting in *in situ* tissue ablation. Presently, cryotherapy represents a minimally invasive alternative treatment for localised or locally advanced prostate cancer. Clinical case series studies confirmed the feasibility of cryotherapy as a primary and salvage treatment for patients with localised or locally advanced prostate cancer (Ismail *et al*, 2007; Cohen *et al*, 2008).

The aim of prostate cryotherapy is to selectively destroy neoplastic tissue and preserve vital structures around the prostate, such as rectum and urinary bladder, and a precise freezing process is required to achieve this goal. The targeted tissue has to be exposed to a temperature lower than −40°C to ensure a complete eradication of the cancer tissue (Larson *et al*, 2000). It is technically challenging to achieve the lethal critical temperature in all the prostatic tissues, especially at the periphery of the ice ball, as this will result in a high percentage of complications. Therefore, complete ablation of prostate cancer tissue often fails and results in local disease recurrence.

Cold injury starts when temperature falls to subzero levels and extracellular ice starts to form at a temperature range between −7 and −20°C. Ice formation will create a hyperosmolar extracellular environment and expose cells to osmotic stress. Lower temperatures (<−15°C) are associated with intracellular ice formation, which is almost always lethal to the cells (Gage and Baust, 1998).

The aquaporin (*AQP*) family of water channels are intrinsic membrane proteins that facilitate selective water and small solute movement across the plasma membrane (Verkman and Mitra, 2000). They were initially characterised in human red blood and renal cells (Preston *et al*, 1992; Nielsen *et al*, 1993). Subsequently, they were identified in mammals, plants, yeasts and arthropods. To date, 13 members of the *AQP* family have been identified in mammals (*AQP0*–*12*). Mammalian *AQPs* were stratified into two subgroups. Members of the first subgroup, including *AQP1*, *AQP2*, *AQP4*, *AQP5*, *AQP6* and *AQP8*, are highly selective for the passage of water across the plasma membrane. The second group is the aquaglyceroporins, which includes *AQP3*, *AQP7*, *AQP9* and *AQP10*; they permit the transport of small non-ionic molecules, such as glycerol, in addition to water (Ishibashi *et al*, 1994).

Only recently, the role of *AQP* in tumour pathogenesis has been identified. Aquaporin 3 was found to be expressed in normal and malignant prostate tissue and may be involved in tumour initiation and development (Wang *et al*, 2007). The expression of *AQP1* is confined to the capillary endothelium of prostate cancer cells and it may be involved in microvascular alteration during tumour angiogenesis (Mobasheri *et al*, 2005). There has been increasing evidence that *AQPs* may play a role in carcinogenesis. The oncogenic properties of *AQP5* in lung and colorectal cancer were examined recently (Woo *et al*, 2008). It was shown that *AQP5* phosphorylation at the PKA substrate consensus site plays a key role in cell proliferation. A recent study by the same group showed that AQP5 expression in colorectal cancer is significantly associated with lung metastasis that is possibly mediated by the activation of Ras, mitogen activated protein kinase (MAPK) and Rb signalling pathways (Kang *et al*, 2008).

In this study, we investigate the effects of inhibiting the *AQP* in sensitising human prostate cancer cells to cryotherapy using an *in vitro* model. The expression of the *AQP* by PC-3 and DU145 cells before and after cryoinjury was determined using qPCR. We have assessed the potential use of RNA interference (RNAi) technology as an adjunctive therapy to cryotherapy to enhance anti-tumour efficacy. To our knowledge, this is the first study that evaluates the role of *AQPs* in the tolerance of human prostate cancer cells to cryoinjury.

## Materials and methods

### Prostate cancer cell culture

The human DU145 and PC-3 cell lines were obtained from the ATCC (American Type Culture Collection). Cells were grown in a complete culture medium (RPMI 1640 with 10% foetal calf serum, 1% L-glutamine and 1% penicillin/streptomycin, all from Sigma-Aldrich, Poole, UK) and incubated at 37°C with 5% CO_2_. Cells were suspended in 5 ml of fresh culture medium. A final cell density of 1 × 10^5^ cells per ml were placed in 1.5 ml Eppendorf tubes and centrifuged at 600 r.p.m. for 1 min.

### Freezing protocol

Cells were treated using the Cryocare system (Endocare Inc., Irvine, CA, USA). Briefly, Eppendorf tubes containing prostate cancer cells were placed 6 mm from the centre of a single cryoprobe and held in place by a temperature probe to monitor the sample temperature. The cells were cooled to 0, −5 and −10°C for 10 min, and then thawed in a 50°C water bath to room temperature. The apparatus allows cells to be treated at a range of temperatures between 0 and −40°C and to be held reproducibly at specific temperatures for as long as required.

### Inhibition of *AQPs* by HgCl_2_

Prostate cancer cells were treated with the *AQP* inhibitor, mercuric chloride (HgCl_2_) (Sigma-Aldrich), at a concentration of 0.075 mM for 15 min. Cells were washed twice and cooled to −10°C for 10 min. Cell survival was compared with the untreated cells, cells cooled in the absence of HgCl_2_ and cells treated with HgCl_2_ only using the 3-(4,5-dimethylthiazol-2-yl)-5-(3-carboxymethoxyphenyl)-2-(4-sulfophenyl)-2H-tetrazolium, inner salt (MTS) assay (Promega, Madison, WI, USA).

### Small interfering RNA (siRNA) synthesis

RNA duplex of 19 nucleotides specific for human *AQP3* sequence was synthesised by Thermo Scientific Dharmacon (Lafayette, CO, USA). ON-TARGET*plus* siRNA (Dharmacon, Lafayette, CO, USA) smart pool is a mixture of four siRNAs targeting *AQP3*, which attains the maximum target gene silencing and reduces the overall number of off-target interactions. ON-TARGET*plus* negative control, which has minimal targeting of known genes in the human genome, was used as a control siRNA.

Target sequence: GGAUCAAGCUGCCCAUCUA
(Sense orientation): CUUCUUGGGUGCUGGAAUA
 UAUGAUCAAUGGCUUCUUU
 GAGCAGAUCUGAGUGGGCA

### Transfection of human prostate cancer cells with siRNA

DU145 and PC-3 cells were diluted in an antibiotic-free complete medium to a plating density of 1.0 × 10^5^ cells per ml and 500 *μ*l of cells were seeded in each well of a 24-well plate. Cells were incubated at 37°C with 5% CO_2_ overnight. Small interfering RNA transfection was carried out using the DharmaFECT 2 transfection reagent (Dharmacon) following the manufacturer's protocol. A stock solution of 2 *μ*M of the *AQP3* siRNA was prepared, aliquoted and stored at −20°C. Aquaporin 3 siRNA was diluted in a ratio of 1 : 1 in a serum-free RPMI medium. In parallel, 3 *μ*l of the DharmaFECT 2 reagent was added to 197 *μ*l of serum-free medium. The two mixtures were combined and incubated for 20 min at room temperature for complex formation. After the addition of a sufficient antibiotic-free complete medium (final concentration of *AQP3* siRNA is 100 nM), 500 *μ*l of the mixture was added to each well and cells incubated at 37°C with 5% CO_2_. Control siRNA was prepared in a similar way to *AQP3* siRNA. Mock-transfected cells are cells that were treated with DharmaFECT 2 reagent only. Cell survival was assessed at day 6 and compared with the untreated cells. Cells were harvested at day 3 for mRNA analysis and at day 6 post transfection for protein analysis.

### Cell treatment after siRNA transfection

*AQP3*-specific gene silencing was confirmed by three independent western blot and q-PCR experiments. Untreated cells, cells treated with control siRNA and mock-transfected cells were used as controls. Transfected cells were harvested and treated with freezing at −10°C as described above. Cell survival was assessed using the MTS assay and compared with the control cells and cells treated at −10°C without transfection.

### Real-time quantitative polymerase chain reaction (RT–qPCR)

Real-time quantitative polymerase chain reaction analysis was performed using the Stratagene Mx3005P qPCR system (Stratagene, La Jolla, CA, USA). Glyceraldehyde 3-phosphate dehydrogenase was used as a reference gene and all reactions were performed in duplicate in a 96-well plate. A volume of 25 *μ*l of PCR reaction mixture contained 1 *μ*l cDNA, 1 *μ*l of each *AQP* primer, 10.5 *μ*l nuclease-free water and 12.5 *μ*l SYBR Green JumpStart Taq ReadyMix (Sigma-Aldrich). Primers were designed using the Primer3 software (http://frodo.wi.mit.edu/) and obtained from the Eurogentec group (Eurogentec Ltd, Southampton, Hampshire, UK). The efficiency of RT–qPCR was calculated using a standard curve. Thermal cycling conditions were as follows: 1 cycle (10 min at 95°C) followed by 40 cycles (30 s at 95°C, 1 min at 60°C and 30 s at 72°C) followed by 1 cycle (1 min 95°C, 30 s at 55°C and 30 s at 95°C). The 2^−ΔΔ*C*T^ method was used to calculate the difference in *C*_T_ value between the treated sample and untreated control and is expressed as a fold change in gene expression relative to the untreated cells. Results were then normalised to an endogenous reference gene (*GAPDH*) whose expression is constant in all groups.

^ΔΔ^*C*T=(*C*_T_,Target−*C*_T_Reference gene)_treated_–(*C*_T_,Target−*C*_T_Reference gene)_untreated_.

### Western blot analysis

After treatment, cells were washed with phosphate-buffered saline (PBS) and lysed with RIPA (RadioImmuno Precipitation Assay) Buffer containing protease and phosphatase inhibitor cocktail and EDTA (ethylenediaminetetraacetic acid) (all from Perbio Science, Cramlington, Northumberland, UK). The cells were then centrifuged at 13 000 r.p.m. for 5 min and the supernatant collected and stored at −20°C. Protein concentration was determined using the BCA protein assay kit (Perbio Science) as per the manufacturer's instructions. A total of 25*μ*g of protein was added to 2.5 *μ*l of sample buffer and 1 *μ*l of reducing agent and incubated at 70°C for 10 min and was then loaded on a Bis Tris gel (all from Invitrogen, Paisley, UK). Proteins were transferred from within the gel onto a PVDF membrane and blocked with 50 ml of blocking buffer (PBS, 0.1% Tween 20 and 5% Bovine serumalbumin). The membrane was incubated with the primary antibody against AQP3 (1 : 1000) (Santa Cruz, Santa Cruz, CA, USA) and *β-actin* (1 : 5000) (Abcam, Cambridge, UK) overnight at 4°C. The membrane was then incubated with a secondary antibody (peroxidase-labelled anti-goat antibody) and detected using the enhanced chemiluminescent detection method (GE Healthcare, Buckinghamshire, UK).

### Immunofluorescence staining

PC-3 and DU145 cells were cooled to −10°C according to freezing protocol and recovered for 24 h in a 37°C and 5% CO_2_ incubator. Cells were washed in PBS, fixed with 10% formalin for 10 min and blocked with the blocking buffer at room temperature for 30 min. The cells were then incubated with the primary antibody against AQP3 (1 : 200 dilution) overnight at 4°C. After washing, the cells were incubated with the secondary Ab (Donkey anti-goat IgG antibody, Alexa Fluor 488 Conjugated, Invitrogen) at room temperature for 1 h. Negative control included cells incubated with the dilution buffer without the addition of a primary antibody to determine the levels of non-specific fluorescence. Immunofluorescence was visualised using Eclipse TE2000-S microscope (Nikon, Japan).

## Results

### Cryotherapy results in increased expression of *AQP3* in prostate cancer cells

**Figure 1:**
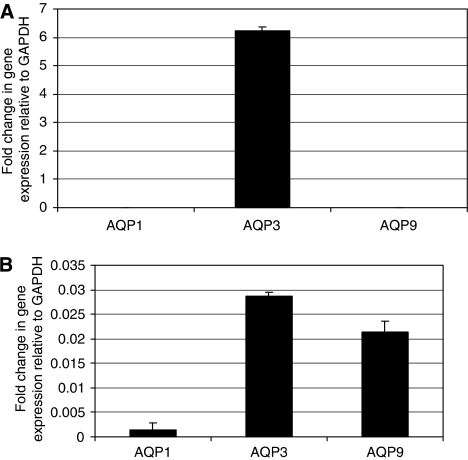
*AQP1*, *AQP3* and *AQP9* mRNA expression in (**A**) DU145 and (**B**) PC-3 human prostate cancer cells. RNA was extracted from fresh cells, reverse-transcribed, and expression levels were calculated as a fold change relative to *GAPDH* expression.

**Figure 2:**
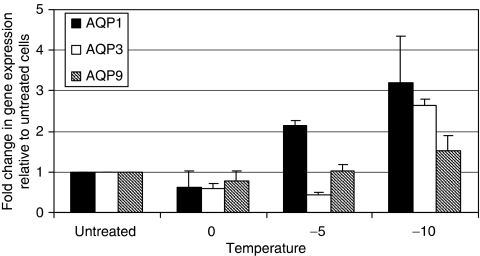
The expression of *AQP1*, *AQP3* and *AQP9* in DU145 cells in response to freezing. Cells were cooled to 0, −5 and −10°C for 10 min and thawed to room temperature. RNA was extracted, reverse-transcribed, and mRNA expression was measured as a fold change relative to the untreated cells. The expressions of *AQP1* and *AQP3* mRNA were significantly increased at −10°C (*P*<0.001).

**Figure 3:**
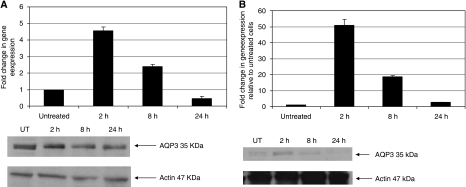
AQP3 expression by RT–qPCR and western blot analysis in (**A**) DU145 and (**B**) PC-3 cells over time intervals post freezing. There was a significant increase in AQP3 expression 2 h after freezing followed by a time-dependent reduction to the control levels.

Prostate cancer cells were cooled to 0, −5 and −10°C for 10 min. After overnight recovery, total RNA and protein were extracted and *AQP1*, *AQP3* and *AQP9* mRNA expression was assessed using q-PCR. The *AQP3* expression in the untreated cells was compared with GAPDH mRNA expression. Untreated DU145 cells expressed significant levels of *AQP3*, which is six-fold more than the expression of *GAPDH* (Figure 1A). Untreated PC-3 cells expressed lower levels of *AQP3* (Figure 1B). There was an overall increase in the expression of *AQP3* in DU145 and PC-3 cells on exposure to −10°C freezing temperature. In DU145 cells, the *AQP3* expression slightly reduced at 0 and −5°C (*P*>0.05) followed by a significant increase in expression at −10°C (*P*<0.001; Figure 2). PC-3 cells showed a similar trend to DU145 cells, with a significant increase in the *AQP3* expression at −10°C compared with the untreated cells (data not shown). The *AQP1* mRNA expression significantly increased in cells cooled to −5 and −10°C (*P*<0.01). The *AQP9* mRNA levels did not change significantly after exposure to cryoinjury. The *AQP3* protein and mRNA expression was evaluated 2, 8 and 24 h post freezing at −10°C using RT–qPCR and western blot analysis. The results show that in the immediate post-freeze period, there are 5- and 50-fold increases in *AQP3* expression in DU145 and PC-3 cells, respectively, followed by a gradual return to the untreated level (Figure 3). This response may represent an important adaptive mechanism used by the cells to face the osmotic stress associated with cryoinjury.

### Inhibition of *AQPs* by mercuric chloride increases the sensitivity of prostate cancer cells to freeze injury

**Figure 4:**
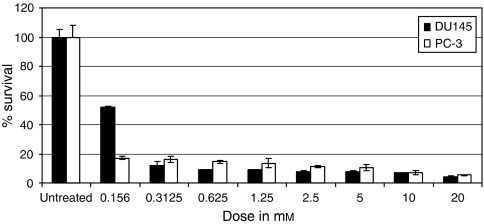
The effect of mercuric chloride (HgCl_2_) on DU145 and PC-3 cell survival. Cells were grown in a 96-well plate and treated with various concentrations of HgCl_2_ in a fresh culture medium for 15 min. Cell survival was assessed using the MTS assay. DU145 cells were more resistant to HgCl_2_ than PC-3 cells.

**Figure 5:**
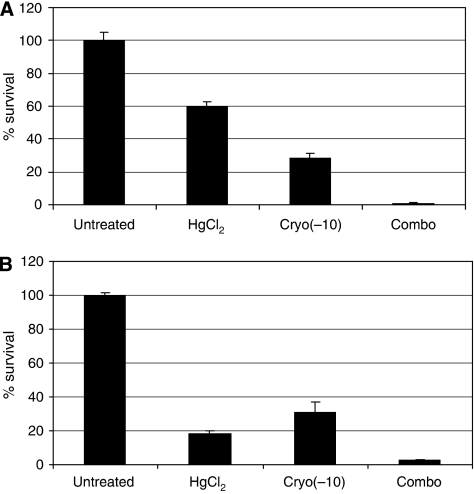
(**A**) DU145 and (**B**) PC-3 cells after exposure to 0.075 mM mercuric chloride (HgCl_2_), freezing (Cryo (−10)) and combination of HgCl_2_ and freezing (Combo). Survival was measured as a percentage of the untreated cells. There was a complete loss of DU145 cell survival at −10°C (*P*<0.001).

Mercuric chloride is an inhibitor of the *AQP*, which has been used routinely to test *AQP* function in animal and plants cells. It has a high affinity to the reactive thiol moiety of cysteine residues within the *AQPs*, causing covalent changes leading to the inhibition of water transport function (Savage and Stroud, 2007). To ascertain the effect of HgCl_2_ on prostate cancer cell survival, DU145 and PC-3 cells were treated with various concentrations of HgCl_2_ for 15 min, and survival was assessed using the MTS assay. PC-3 cells were very sensitive to HgCl_2_ treatment and more than 80% died at low concentration (0.075 mM). DU145 cells were more resistant to HgCl_2_ and the IC_50_ was 0.15 mM (Figure 4). DU145 cells were treated with 0.078 mM of HgCl_2_ for 15 min, and the cells were then cooled to −10°C for 10 min. Cell survival was assessed using the MTS assay and compared with the untreated cells, cells treated with HgCl_2_ or freezing alone. Mercuric chloride or cryotherapy treatment alone resulted in a 40 and 80% reduction in cell survival, respectively. The combination treatment resulted in a significant reduction in cell survival compared with either treatment alone, with almost a complete loss of cell viability after treatment (*P*<0.001; Figure 5). To determine whether the combination treatment is synergistic, the combination index (CI) was calculated using CalcuSyn software (Biosoft, Cambridge, UK) that uses the median effect principle. The CI provides a quantitative measure of the degree of interaction between two or more agents (Chou and Talalay, 1984). The CI for the combination treatment was 0.2, which denotes a marked synergy between the two treatments.

### *AQP3* inhibition by siRNA in prostate cancer cells

**Figure 6:**
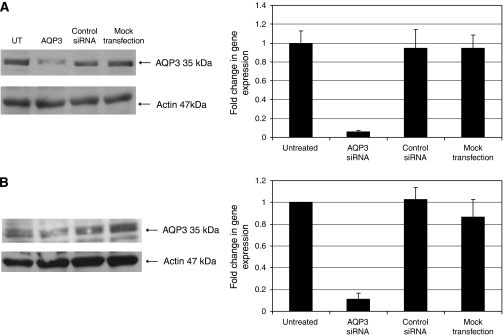
*AQP3* mRNA and protein levels in (**A**) DU145 and (**B**) PC-3 cells were assessed by RT–qPCR and western blot analysis 3 and 6 days after *AQP3* transfection, respectively. Multiple controls were used to verify the specificity of the treatment.

To further investigate the role of specific *AQPs* in protecting prostate cancer cells from cryoinjury, an *AQP3* silencing experiment was carried out with 100 nM of *AQP3* siRNA. Aquaporin 3 gene silencing was monitored by RT–qPCR at day 3 and western blot analysis at day 6 after inducing RNAi. A control siRNA was used in parallel to test for the potential non-specific effects of the short RNA duplex. To evaluate the toxic effect of transfection on overall viability, cell survival was assessed using the MTS assay at day 6 after the addition of the siRNA. The percentage of cell survival was above 80% in all groups indicating optimal transfection conditions with no toxic effect of the lipid complex on the treated cells (*P*>0.05; data not shown). The results show that *AQP3* mRNA levels were significantly decreased 3 days after the treatment with siRNA to <10% of the control. There was no significant change in the *AQP3* mRNA expression in cells treated with control siRNA and mock-transfected cells. Western blot analysis showed a reduction in *AQP3* protein levels at day 6, whereas protein expression levels were unaffected in cells treated with control siRNA and in mock-transfected cells showing the specificity of the results (Figure 6).

### Assessment of cell survival in *AQP3* knockdown cells compared with normal cells

**Figure 7:**
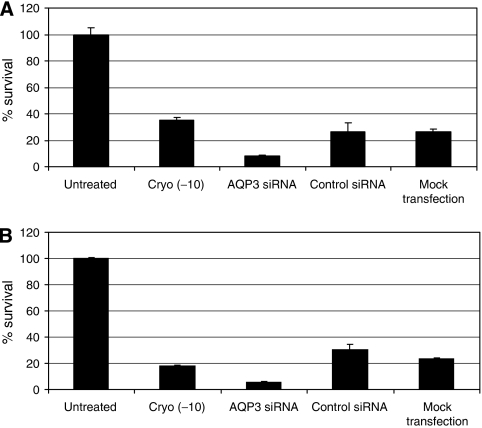
(**A**) DU145 and (**B**) PC-3 cell after exposure to cryoinjury. *AQP3* knockdown cells showed a significant reduction in cell viability after exposure to cryoinjury compared with the other groups (*P*<0.001). Cell survival is expressed as a percentage of the untreated cells.

The *AQP* inhibitor (HgCl_2_) resulted in a significant decrease in DU145 cell viability after exposure to −10°C. To further investigate the specificity of *AQP3* inhibition in increasing cell sensitivity to cryotherapy, different cell groups were cooled to −10°C and cell viability was assessed using the MTS assay. Four groups were compared, namely fresh cells (cryo −10), cells transfected with *AQP3* siRNA (*AQP3* siRNA), cells transfected with control siRNA (control siRNA) and mock-transfected cells (mock transfection). Cell survival was assessed as a percentage relative to the untreated cells. The results show a statistically significant increase in cell death after exposure to cryoinjury in *AQP3* knockdown cells compared with fresh cells (*P*<0.001; Figure 7). Cells treated with control siRNA and mock-transfected cells showed a similar cell viability to fresh cells after exposure to freezing (*P*>0.05). We showed clearly that increased sensitivity to cryoinjury was specific to *AQP3* knockdown in DU145 and PC-3 cells indicating that the *AQP3* gene product may play a role in protecting prostate cancer cells from cryoinjury.

### Cryotherapy results in relocalisation of *AQP3* into the plasma membrane

**Figure 8:**
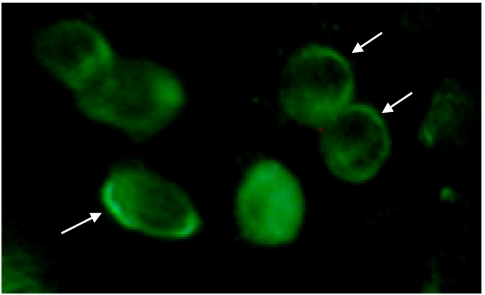
Immunofluorescence expression and localisation of AQP3 post cryotherapy. It is clear that AQP3 is expressed on the plasma membrane of PC-3 cells. A similar finding was observed in DU145 cells. Original magnification × 600.

It was shown that *AQP3* protein is expressed in the cytoplasm of the human prostate cancer cells (Wang *et al*, 2007). Transporter proteins need to be expressed in the plasma membrane to function. In this study, we investigated the expression and localisation of *AQP3* in prostate cancer cells in response to cryoinjury using immunofluorescence staining. Cryotherapy markedly redistributed *AQP3* protein into the plasma membrane of prostate cancer cells. Figure 8 clearly shows membrane expression of *AQP3* in PC-3 cells. DU145 cells showed a similar finding (data not shown). Control wells incubated with dilution buffer showed no immunoreactivity (data not shown).

## Discussion

Cryotherapy is an effective therapy for localised or locally advanced prostate cancer (Ismail *et al*, 2007). Clinical reports indicate that −40°C represents the ideal target temperature for a satisfactory prostate tissue ablation (Larson *et al*, 2000). As this is not achieved clinically, a complete ablation of cancer tissue some times fails and results in disease recurrence. Therefore, a second synergistic therapy is of great potential benefit. Some synergistic cell killing has been achieved with concomitant cryotherapy and chemotherapy, radiotherapy, hyperthermia and apoptosis inducing ligands (Robilotto *et al*, 2007).

In this study, we identified inhibition of *AQPs* as a further therapeutic target that may be synergistic with cryotherapy. During the initial phase of freezing, ice formation starts in the extracellular space creating a hyperosmolar environment and leading to water escape from the intracellular to the extracellular space along the osmotic gradient. Intracellular hyperosmolarity will result in a reduction in the critical temperature of intracellular ice formation. Inhibiting the *AQPs*, which are primarily responsible for controlling water transport across the plasma membrane, will lead to intracellular water retention and to a subsequent increase in the critical temperature at which intracellular ice will form.

In this study, we provide strong evidence that *AQP* protein can play a key role in modulating prostate cancer cell response to cryotherapy. Aquaporin 3 was expressed in both prostate cancer cell lines and it was used as a target in this study. Exposure to freezing temperature provided a strong stimulus for enhanced expression of *AQP3* in an attempt to overcome the osmotic stress. Similar upregulation on exposure to mild hypothermia was reported in earlier studies (Fujita *et al*, 2003). Cryotherapy increases the expression of *AQP3* through two mechanisms. First, by creating a hyperosmolar environment at the extracellular space that leads to increased expression of *AQP3* (Bell *et al*, 2009). Second, we found a five-fold increase in *MAPK14* expression in prostate cancer cells on exposure to −10°C freezing temperature (unpublished data). Bell *et al* showed that *MAPK14* is involved directly in regulating *AQP3* expression in response to hyperosmotic stress. Therefore, the upregulation of *AQP3* can be considered as part of a protective physiological stress response to the osmotic changes at the initial phase of cryoinjury. Aquaporin 3 is a transporter protein that needs to be expressed in the cell membrane to function. Wang *et al* showed that *AQP3* is expressed in the cytoplasm of prostate cancer cells. On the basis of our finding from immunofluorescence staining, we showed clearly that cryoinjury results in the relocalisation of AQP3 from the cytoplasm to the plasma membrane, which may reflect the direct involvement of *AQP3* in the intracellular osmotic changes associated with cryoinjury.

Earlier studies have reported on the relationship between *AQP* water channels and freeze tolerance. Forced overexpression of *AQP3* in mouse oocytes increased survival after cryopreservation in a glycerol-based medium (Edashige *et al*, 2003). Gene expression analysis in baker's yeast showed a correlation between freeze resistance and the expression of *AQP1* and *AQP2* genes (Tanghe *et al*, 2002). Deletion of the respective genes has raised the sensitivity to cryoinjury. It was also shown that the high level of freeze tolerance in arthropods was significantly reduced when the expression of the *AQP* was inhibited by HgCl_2_ even in the presence of glycerol as a cryoprotectant (Izumi *et al*, 2006; Philip *et al*, 2008).

On the basis of these observations, we examined the role of the *AQP* in freeze tolerance of human prostate cancer cells. DU145 cells and PC-3 cells were frozen in the presence or absence of mercuric chloride, a universal inhibitor of the *AQPs* (Niemietz and Tyerman, 2002). We showed that cells exposed to combined treatment were more seriously damaged by freezing at −10°C compared with cells exposed to cryotherapy alone. Inhibition of the *AQP* interferes with water transport across the plasma membrane down the osmotic gradient, which is essential for maintaining the osmotic equilibrium and freeze tolerance (Philip *et al*, 2008). Izumi *et al* (2006) suggested that freezing stress requires control of water and solutes across the plasma membrane and cell survival will depend on the portion of intracellular water replaced by cryoprotectant. In this study, our aim was to increase the efficacy of cryotherapy, therefore no cryoprotectant was used.

To further characterise the role of the *AQP* in freeze tolerance in human prostate cancer cells, RNAi technology was used to knock down *AQP3* protein expression. We showed that although silencing of *AQP3* protein did not affect cell proliferation (data not shown), it resulted in increased sensitivity of cancer cell to cryoinjury compared with untreated cells. These data provide compelling evidence that expression of the *AQP* on the plasma membrane is essential for protecting human prostate cancer cells from cryoinjury, suggesting a specific functional role of the *AQP* in cancer cell biology. Thus, *AQP* inhibition by pharmacological blockers might provide a new therapeutic approach for sensitising human prostate cancer cells to cryotherapy.
