# Inhibition of the aquaporin 3 water channel increases the sensitivity of prostate cancer cells to cryotherapy

**DOI:** 10.1038/sj.bjc.6605233

**Published:** 2009-07-28

**Authors:** M Ismail, S Bokaee, R Morgan, J Davies, K J Harrington, H Pandha

**Affiliations:** 1 Department of Oncology, Postgraduate Medical School, University of Surrey, Guildford GU2 7WG, UK; 2 Department of Urology, The Royal Surrey County Hospital, Guildford GU2 7XX, UK; 3 Targeted Therapy Laboratory, The Institute of Cancer Research, 237 Fulham Road, London SW3 6JB, UK

**Correction to**: *British Journal of Cancer* (2009) **100,** 1889–1895; doi:10.1038/sj.bjc.6605093

Owing to an error during typesetting, the name of the third author, Richard Morgan, was omitted from this paper when first published in the June 2009 issue of BJC. The publishers would like to apologise for this mistake.

